# How to quantify plant tolerance to loss of biomass?

**DOI:** 10.1002/ece3.2907

**Published:** 2017-03-28

**Authors:** Tom J. de Jong, Tiantian Lin

**Affiliations:** ^1^ Institute of Biology LeidenLeidenThe Netherlands; ^2^ College of ForestrySichuan Agricultural University611130, ChengduChina

**Keywords:** herbivory, invasive plants, regrowth, shoot–root ratio

## Abstract

In some plant species the whole shoot is occasionally removed, as a result of specialist herbivory, grazing, mowing, or other causes. The plant can adapt to defoliation by allocating more to tolerance and less to growth and defense. Plant tolerance to defoliation (TOL1) is typically measured as the ratio between the average dry weight of a group of damaged plants and a control group of undamaged plants, both measured some time after recovery. We develop a model to clarify what TOL1 actually measures. We advocate keeping regrowth (REG2) and shoot–root ratio, both elements of TOL1, separate in the analysis. Based on a resource trade‐off, exotic *Jacobaea vulgaris* plants from populations in the USA (no specialist herbivory) are expected to grow faster and be less tolerant than native Dutch populations (with specialist herbivory). Indeed Dutch plants had both a significantly larger fraction biomass in roots and faster regrowth (REG2), while US plants attained the highest weight in the control without defoliation. Using key‐factor analysis, we illustrate how growth rates, regrowth, and shoot–root ratio each contribute to final biomass (plant fitness). Our proposed method gives more insight in the mechanisms that underly plant tolerance against defoliation and how tolerance contributes to fitness.

## 1 Introduction

Specialist herbivores typically adapt to the defense chemicals of their preferred food plant (Crawley, 1983). This makes chemical defense ineffective, and the last resort of the plant is to develop tolerance, the ability to regrow after some level of defoliation (McNaughton, 1983). Plant species that invade a new area or continent escape, for some time at least, from their specialist herbivores. Natural selection may then lead to a shift in allocation patterns; adaptation to the new environment could reduce allocation to tolerance and increase allocation to growth and defense against generalist herbivores (Bossdorf et al., 2005; Keane & Crawley, 2002; Vrieling & Joshi, 2005). Do plants adapt to their new environment and in what way?

Reciprocal transplant experiments provide the most direct test of local adaptation. Such transplants date back to the classic work of Clausen, Keck, and Hiesey (Núñez‐Farfán & Schlichting, 2001) in the first part of the 20th century and are still highly relevant today. Some modern studies (Genton, Kotanen, Cheptou, Adolphe, & Shykoff, 2005) transplanted plants over continents, following their recent range expansion. The home genotype is expected to produce most seeds or biomass, that is, attain the highest fitness. A subsequent question is which physiological adaptations allow for the success of the home genotype? To answer this question in the context of tolerance, a typical experiment has been designed (Belsky, 1986; Bustos‐Segura, Fornoni, & Nunez‐Farfan, 2014; Jogesh, Stanley, & Berenbaum, 2014; Scholes, Wszalek, & Paige, 2015; Strauss & Agrawal, 1999). This involves placing different genotypes into a common environment (growth room) and after some time apply complete defoliation to half the plants, while leaving the other half untouched. Under the rather stringent assumption that the artificial defoliation treatment is representative of natural defoliation, one expects a native genotype to perform best in the defoliation treatment. When an exotic genotype allocates less to tolerance and more to growth, it is expected to outperform a native genotype in the control treatment. In such experiments, fitness is the number of seeds or biomass at the end of the experiment.

In analyzing these defoliation experiments, researchers have developed several measures of tolerance. The most commonly used tolerance measure is the degree to which final biomass is affected by herbivore damage, relative to the undamaged state (TOL1, Strauss & Agrawal, 1999). This tolerance measure can be compared between different genotypes but does not necessarily reflect fitness. Suppose genotype A produces 50 seeds when undamaged and 40 when damaged and genotype B produces 100 seeds when damaged and 50 when undamaged. Then in the damage treatment, genotype B has the highest fitness (50 seeds produced by B, 40 seeds by A), despite its low tolerance (TOL1 = 0.5 for B, 0.8 for A). It would be incorrect to state that A has higher tolerance and is therefore better adapted to an environment with frequent herbivory. This distinction between fitness and tolerance was already pointed out clearly by Hochwender, Marquis, and Stowe (2000).

**Figure 1:**
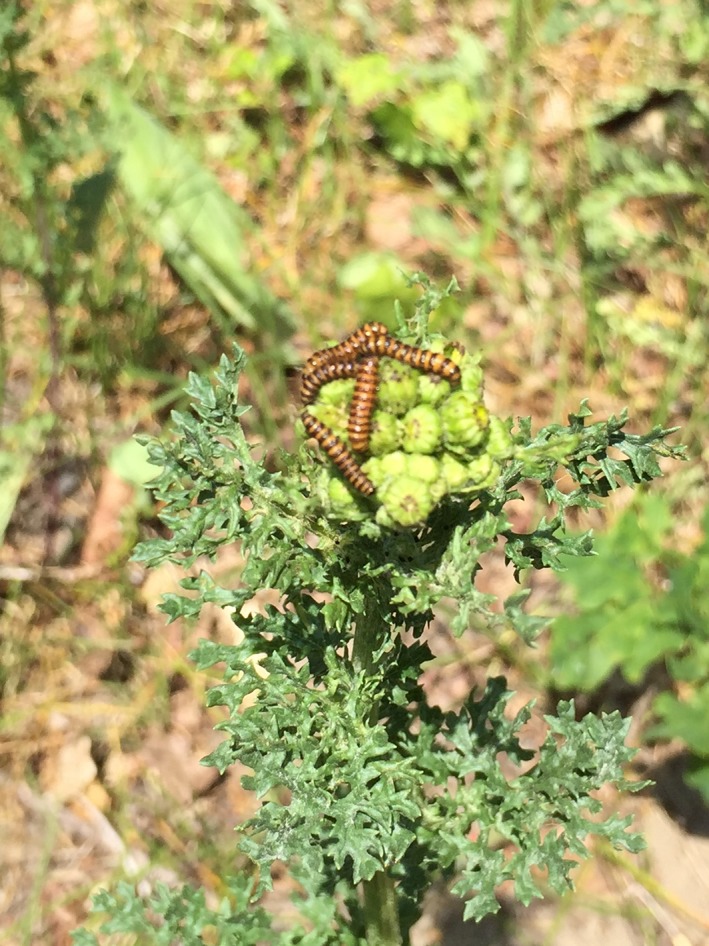
The plant species *Jacobaea vulgaris* (ragwort) is frequently defoliated by its specialized herbivore *Tyria jacobaeae* (the cinnabar moth). After complete defoliation, the plant can recover by forming new rosettes at the stem or from root fragments. These new rosettes may flower in the subsequent year. Photograph T. Lin

Several studies compared tolerance of native and exotic plants. In *Pastinaca sativa*, tolerance to webworm herbivory increased after introduction of a specialized herbivore in Australia (Jogesh et al., 2014). *Jacobaea vulgaris* genotypes from native populations (Europe) with regular defoliation by the specialist *Tyria jacobaeae* (Bonsall, van der Meijden, & Crawley, 2003) had higher regrowth capacity than genotypes from populations in the USA or Australia without such defoliation (Vrieling & Joshi, 2005; Figure 1). Contrary to this, tolerance was higher in exotic US populations of the tree *Sapium sebiferum*, as compared to native Chinese populations (Zou, Rogers, & Siemann, 2008). For the subtropical shrub *Chromolaena odorata*, no difference in the tolerance measure was found between native populations from the south of the USA and Mexico and invasive Chinese populations (Li, Feng, & Barclay, 2012).

The greatest value of tolerance measures is, in our opinion, to clarify the mechanisms behind tolerance. Two plant strategies result in high values for TOL1. First, plants can reduce the fraction biomass lost through defoliation by storing more resources belowground, that is, a low shoot–root ratio (Hochwender et al., 2000; Li et al., 2012; Stowe, Marquis, Hochwender, & Simms, 2000; van der Meijden, de Boer, & van der Veen‐van Wijk, 2000). Second, fast regrowth after defoliation contributes to tolerance.

In this study, we use a simple model of exponential plant growth to illustrate how TOL1 and other tolerance measures depend on both shoot–root ratio and regrowth. Next, we advocate a new measure (REG2) for regrowth, which does not depend on shoot–root ratio. We illustrate how to compute REG2, using a small dataset of *J. vulgaris* that includes native and exotic genotypes. With key‐factor analysis, we show how fitness (the final dry mass of plants) can be related to separate components of tolerance (REG2 and shoot–root ratio) and to other plants characters.

## 2 A Graphical Model

**Figure 2:**
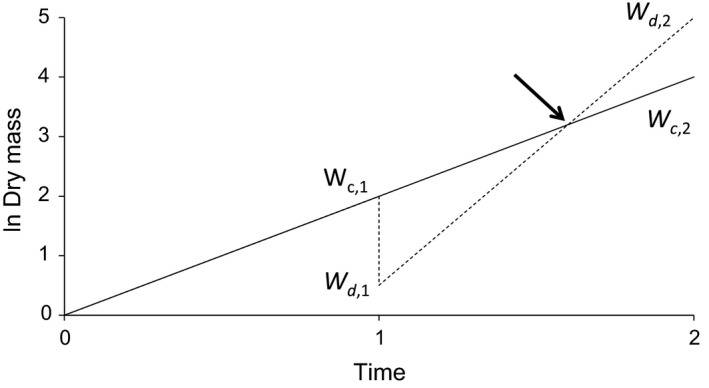
Plants grow exponentially over time. Then at *t* = 1, one group of plants is damaged (*d*) by removing the entire shoot (broken line). This removes a fraction *h* of the biomass and only the root remains, holding a fraction 1 − *h* of the biomass. These plants regrow at a certain rate. A group of control plants (c) is not defoliated (solid line). Because the damaged plants grow faster than the control plants (compensation), they eventually catch up, indicated by the arrow. Overcompensation occurs at the right of this point.

At some time, the whole shoot is removed experimentally. The genotype that produces most seeds at the end of the experiment has the highest fitness. When the damaged plant regrows faster than the control, this is called compensation (Figure 2). Compensation may be due to increased photosynthesis, increased nutrient uptake, a different growth form, allocating the stored resources in the root back to the shoot, and switching defense pathways on or off (reviewed in Rosenthal & Kotanen, 1994). Overcompensation occurs when, after some time, the weight of damaged plant exceeds that of the control (indicated by the arrow in Figure 2, Strauss & Agrawal, 1999).

**Figure 3:**
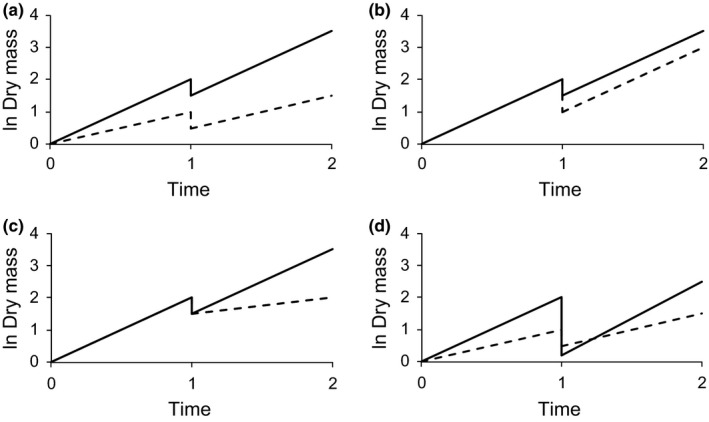
Four different strategies to cope with defoliation. Plants begin to grow at *t* = 0 are completely defoliated at *t* = 1 and then regrow until *t* = 2. Two genotypes A and B are depicted, solid and broken lines. In all cases, the strategy depicted by the solid line wins (highest dry mass at *t* = 2), but for different reasons. (a) Strategy A wins because of its higher RGR. (b) Genotype A wins because its greater storage in roots reduces the fraction biomass lost. (c) Genotype A wins because has higher regrowth. (d) Of course many combinations are possible. In this case, genotype A has fast growth and a high shoot/root ratio. Consequently, it grows faster but suffers more from removal of the whole shoot. At time 2, type A wins, but with earlier harvest, it would not have enough time to catch up and would lose

Plants can deal with biomass removal in different ways. The first strategy is simply to have a high relative growth rate (RGR) throughout, even without specific adaptations for regrowth (Figure 3a). Even if the fast‐growing genotype suffers most after a single defoliation, it will eventually catch up with a slower‐growing genotype and win (Figure 3d). The second strategy could be to have a low shoot–root ratio, which reduces the fraction biomass lost (Figure 3b). Storage in the root, in the form of inulin or starch, draws resources away from photosynthesis and growth. But this can still be a winning strategy when there is limited time after defoliation and the fast grower cannot catch up (Figure 3d, de Jong & van der Meijden, 2000). The third strategy is compensatory regrowth (Figure 3c), as detailed above.

## 3 A Mathematical Model

With exponential growth, it is convenient to plot the weight of the plant on a log scale (Figure 1). The slope corresponds to the RGR (in gram per gram per time unit, all parameters are summarized in Table 1). Plants start to grow at *t* = 0 are completely defoliated at *t* = 1 and then regrow until the experiment is finished, and seeds or biomass are measured, at *t* = 2. We assume exponential growth at a constant rate and, for simplicity, that the time interval between *t* = 0 and *t* = 1 equals that between *t* = 1 and *t* = 2. Uncut control plants have subscript *c*. Damaged plant have subscript *d*. Before defoliation weight of all plants is multiplied by a factor λ_1_ between *t* = 0 and *t* = 1. The weight of control plants is multiplied by a factor λ_c,2_ between *t* = 1 and *t* = 2. Damaged plants grow with a factor λ_d,2_ in that same period. RGRs are the natural logarithms of the λ's (RGR = ln λ or λ = *e* ^RGR^).

**Table 1:** Summary of parameters

Parameter	
*h*	Fraction biomass removed, as the whole shoot is removed this equals the fraction biomass in the roots
*W*	Dry mass in g
d, c	Subscript used to distinguish damaged (*d*) and undamaged control (*c*) plants
RGR (see Equation (1a), (1b))	Relative growth rate in g per g per week
REG1 (see Equation 2)	Regrowth measure 1 comparing RGR of damaged plants in period 2 (after defoliation) with their RGR in period 1 (before defoliation). Unit g per g per week
REG2 (Equations 3, and 4)	Regrowth measure 2 comparing RGR of damaged plants in period 2 (after defoliation) with the RGR of undamaged control plants in the same period. g per g per week. Recommended
TOL1 (Equation 5)	Ratio of dry mass of damaged and control plants at the end of the experimental period. TOL1 is commonly used in the literature. g per g
TOL2	Difference of dry mass of damaged and control plants at the end of the experimental period. Unit g
TOL3	Dry mass of the shoot of damaged plants at the end of the experiment, divided by the shoot dry mass removed at defoliation. Unit g per g

We then have for the control plants:

(1a) $$W_{c,1}=\lambda_{1}W_{0}\;\text{and}\;W_{c,2}=\lambda_{c,2}W_{c,1}.$$

At defoliation, a fraction *h* is removed and a fraction 1 − *h*, the biomass in the root, remains. The weight of the defoliated plants at *t* = 1 is then a fraction 1 − *h* of the control plants just before defoliation occurs. The plants subsequently regrow until *t* = 2 with a factor λ_d,2_. In equation

(1b) $$W_{d,1}=\left(1-h\right)W_{c,1}\;\text{and}\;W_{d,2}=\lambda_{d,2}W_{d,1}.$$

In Figure 2, plant weight is plotted on a log scale, so that the slope of the line for one time unit (i.e., the RGR) before the defoliation is ln(*W*_c,1_) − ln(*W*_0_). In the second period, control plants have a RGR of ln(*W*_c,2_) − ln(*W*_c,1_) and when exponential growth continues at the same rate, this equals the RGR in the first period. The RGR for damaged plants in the second period is ln(*W*_d,2_) − ln(*W*_d,1_).

## 4 Proposed Regrowth Measures

### 4.1 REG1

An intuitive way to define regrowth (REG) is to compare the RGR in the first period and in the second period after damage. The higher this number (or less negative), the higher the regrowth. If there is no control that remains undamaged throughout the experiment, the only option is to compare RGR after damage in the second period to RGR in the first period before damage occurs:

(2) $$\text{REG}1={\text{RGR}}_{d,2}-{\text{RGR}}_{c,1}=\ln\left(\lambda_{d,2}\right)-\ln\left(\lambda_{1}\right)=\ln\left(\frac{W_{d,2}}{W_{d,1}}\right)-\ln\left(\frac{W_{c,1}}{W_{0}}\right).$$

### 4.2 REG2

If the experimental setup includes an undamaged control that continues growing until *t* = 2, one could compare RGR of damaged plants in the second period with RGR of the control in that same period. This gives

(3) $$\text{REG}2={\text{RGR}}_{d,2}-{\text{RGR}}_{c,2}=\ln\left(\lambda_{d,2}\right)-\ln\left(\lambda_{c,2}\right)=\ln\left(\frac{W_{d,2}}{W_{d,1}}\right)-\ln\left(\frac{W_{c,2}}{W_{c,1}}\right).$$

Using Equation 1b, this can be rewritten as

(4) $$\text{REG}2=\ln\left(\frac{W_{d,2}}{\left(1-h\right)W_{c,2}}\right).$$

Equation 4 for regrowth ability makes sense as the numerator gives the measured final weight of the defoliated plants. A fraction 1 − *h* of their original weight remained after defoliation and if the plants would continue at exactly the same rate as before, they would reach a final weight that is a fraction 1 − *h* of the weight of the undamaged controls. In that case (Figure 2b), REG2 is zero. If the RGR of the damaged plants is slower than RGR of the control plants in the second period, then REG2 is negative. If RGR of damaged plants is larger that of controls, then REG2 is positive and there is compensation (Figure 2); with enough time the weight of the damaged plants will exceed that of the control (overcompensation).

## 5 Tolerance Measures Used

Following Strauss and Agrawal (1999) tolerance is typically measured as the ratio (see also Joshi & Vrieling, 2005; van der Meijden et al., 2000) of the weight of the damaged plant (*W*_d,2_) and control plant (*W*_c,2_). This tolerance measure is (using Equation (1a), (1b))

(5) $$\text{TOL1}=\frac{W_{d,2}}{W_{c,2}}=\frac{\lambda_{d,2}\left(1-h\right)W_{c,1}}{\lambda_{c,2}W_{c,1}}=\left(1-h\right)\frac{\lambda_{d,2}}{\lambda_{c,2}}.$$

Thus, TOL1 includes the ratio of the growth factors of the damaged and control plants λ_d,2_/λ_c,2_, which is similar to Equation 2 in which we took the logarithm of this ratio. However, TOL1 also includes 1 − *h*, the fraction biomass in the roots (Equation 5). Both are separate aspects of tolerance. Thus, if TOL1 is higher for group A than for B, this could be either because plants in group A have better regrowth, or because they have a high fraction biomass in roots (reducing the fraction biomass lost through defoliation). If TOL1 is not different, it could mean that there are no differences between the groups, neither in the fraction biomass in roots nor in regrowth. But it could also mean that differences in fraction biomass in roots and regrowth go in different directions that cancel out when we calculate TOL1.

When taking the difference between *W*_d,2_ and *W*_c,2_ as a tolerance measure (TOL2), instead of the ratio as in Equation 5, *W*_c,1_ no longer cancels from the equation. This means that also higher growth in the initial period before biomass removal would increase the score for this measure. This makes interpretation of the tolerance measure more complex.

Lin, Klinkhamer, and Vrieling (2015) divided shoot of damaged plants at *t* = 2 by shoot of control plants at *t* = 1 and used this variable as a tolerance measure. Then,

$$\text{TOL3}=\frac{W_{d,2}}{W_{c,1}}=\frac{\lambda_{d,2}\left(1-h\right)W_{c,1}}{W_{c,1}}=\left(1-h\right)\lambda_{d,2}.$$

A genotype scores high for tolerance measure TOL3 when it has a high growth rate after damage in absolute sense (not in comparison with an undamaged control) and when it has low shoot–root ratio so that the fraction biomass removed is low.

All tolerance measures depend in slightly different ways from both regrowth, the fraction biomass in roots or even the RGR. This problem can be avoided by calculating REG2 and the fraction biomass in roots separately. This is illustrated in the next paragraph.

## 6 Data on Tolerance of *Jacobaea vulgaris*

Seeds of *J. vulgaris* from three different mother plants of five native populations (the Netherlands) and six exotic populations (USA) were germinated (Lin, 2015). In Dutch populations, the specialist *Tyria jacobaea* regularly defoliates plants (Bonsall et al., 2003), while this herbivore is absent in the US populations. It is expected that native Dutch genotypes invest in tolerance and perform best in a defoliation treatment. The US genotypes may invest more in growth and would then grow best in the control treatment without defoliation. Four well‐grown seedlings from each mother plant were selected and randomly assigned to four groups. For each group, there were 1 seedling × 3 motherplants × 11 populations = 33 plants. The first group was harvested before potting, and dry mass of seedlings was measured. The remaining groups were allowed to grow in 1‐L pots with 20% potting soil (Slingerland potting soil, Zoeterwoude, the Netherlands), 80% sandy soil (collected from Meijendel, the Netherlands, 52°13′N, 4°34′E), and 2.5 g Osmocote slow‐release fertilizer (Scott^®^, Scotts Miracle‐Gro, Marysville, OH, USA; N:P:K:MgO 15:9:11:2.5). Plants were grown in a climate room at 20°C, 70% humidity, 16 hr daylight with a light intensity of 113 μmol PAR m^−2^ s^−1^. After 8 weeks of growth, plants from the second group were harvested to estimate the fraction dry mass in shoot and root. Meanwhile, the whole shoot was removed for the 33 plants in the third group, while the last group was undamaged. These two groups were allowed to grow for another 4 weeks before all plants were harvested. The data in Table 2 are always the averages of three plants from the same population.

**Table 2:** Averages of growth parameters from five Dutch^a^ and six US^b^ populations of *Jacobaea vulgaris* that were allowed to grow in period 1, then defoliated and were allowed to regrow in period 2

Collecting site	*W*_d,2_ (g) fitness	Fraction roots	RGR_c,1_ (I)	RGR_c,2_ (II)	RGR_d,2_ (III)	REG1 III–I	REG2 III–II	TOL1^c^
Meijendel	2.201	0.361	0.894	0.392	0.306	−0.588	−0.086	0.256
Wageningen	2.117	0.322	0.863	0.366	0.388	−0.474	+0.022	0.352
Mossel	1.408	0.398	0.918	0.249	0.123	−0.795	−0.126	0.240
Gees	1.622	0.385	0.921	0.225	0.159	−0.762	−0.065	0.297
Texel	1.688	0.416	0.885	0.274	0.223	−0.661	−0.050	0.340
Indian Creek, OR	1.755	0.326	0.908	0.365	0.230	−0.679	−0.136	0.189
West Crest M, OR	1.380	0.260	0.950	0.243	0.143	−0.807	−0.100	0.174
Island Lake, OR	1.552	0.229	0.873	0.410	0.359	−0.514	−0.052	0.186
Little Wolf, MT	1.050	0.273	0.897	0.354	0.168	−0.729	−0.186	0.130
Kootenai, MT	1.432	0.272	0.918	0.401	0.205	−0.713	−0.196	0.124
Cochran Creek, OR	1.146	0.280	0.870	0.384	0.237	−0.633	−0.147	0.156
Average (*SE*) NL	1.807 (.151)	0.377 (.016)	0.896 (.011)	0.301 (.032)	0.240 (.048)	−0.656 (.041)	−0.061 (.024)	0.297 (.022)
Average (*SE*) US	1.386 (.105)	0.273 (.012)**	0.902 (.012)	0.360 (.024)	0.224 (.031)	−0.679 (.041)	−0.136 (.022)*	0.160 (.011)**
	*p* = .054	*p* = .001	*p* = .700	*p* = .196	*p* = .786	*p* = .754	*p* = .049	*p* = .001

a Initial weight of all Dutch plants 0.0014 g.

b Initial weight of all US plants 0.0015 g.

c DW damaged plants at *t* = 2/DW undamaged plant at *t* = 2.

*p ≤ .05, **p < .01, Welch *t* test.

These results can be summarized as follows. In the selective regime of defoliation the Dutch plants attained the highest dry mass; *W*_d,2_ = 1.807 g for the Dutch and 1.386 g for the US plants (Table 2). Without damage, the US control plants tended to grow faster than the Dutch plants, the final dry weight at *t* = 2 was 41% higher (result not shown), even though the RGRs were not significantly different between Dutch and US plants in neither period 1 nor period 2 (Table 2). The results are consistent with our hypothesis that Dutch plants invest relatively more in tolerance and US plants in growth. Plants from Dutch populations had a significant higher fraction from their biomass in roots than plants from North America (Table 2, Welch *t* test, *p* = .001) and therefore suffered relatively less biomass loss. The RGR of the damaged plants was higher for Meijendel plants, but not significantly. REG1 showed no difference between Dutch and US populations. REG2 was less negative for the Dutch plants showing that they regrow better (Welch *t* test, *p* = .049). This occurs because the RGR of the damaged plants was higher for the Dutch populations and the RGR for the undamaged plants was smaller. One of the Dutch populations even showed a positive value for REG2, indicating compensation. TOL1 is highly significantly different between Dutch and US plants (Welch *t* test, *p* = .0014), reflecting differences in both fraction roots and REG2. Table 1 shows that RGR of the control plants in the first period is over twice as high as in the second period. Apparently some resource became limiting as these plants grew larger or self‐shading occurred.

## 7 Connecting Plant Parameters to Dry Mass (Fitness)

Under the damage regime used, fitness is the final weight of the damaged plants, *W*_d,2_. Plants grew 8 weeks, were then cut, and regrew for 4 weeks. In equation

(6) $$W_{d,2}=W_{0}\lambda_{1}^{8}\left(1-h\right)\lambda_{d,2}^{4}.$$

We defined REG2 as RGR_*d*,2_ − RGR_*c*,2_ so that RGR_*d*,2_ = REG2 + RGR_*c*,2_. Substituting this in Equation 6, using λ = *e* ^RGR^ and taking natural logarithms gives

(7) $$\ln\left(W_{d,2}\right)=\ln\left(W_{0}\right)+8{\text{RGR}}_{c,1}+\ln\left(1-h\right)+4{\text{RGR}}_{c,2}+4\text{REG}2.$$

Equation 6 subdivides the log of fitness into the log of initial weight, the RGR in the first 8 weeks, the log of the fraction in dry mass in roots that escapes herbivory, the RGR of undamaged control plants and the regrowth capacity REG2. We can regress all different components (*c*_*i*_) on total fitness to estimate their relative effect, *c*_*i*_ = *a* + β_*i*_ ln(*W*_d,2_). This procedure is called key‐factor analysis and Royama (1996) discussed this specific method. The value of β gives the relative importance of a factor for the variation in the logarithm of some multiplicative factor. This multiplicative factor is usually survival, but in this case, it is fitness, including both growth and biomass lost. The five values of β_*i*_ sum to 1, so that it is possible to compare the relative effects of different stages on ln(*W*_d,2_).

**Figure 4:**
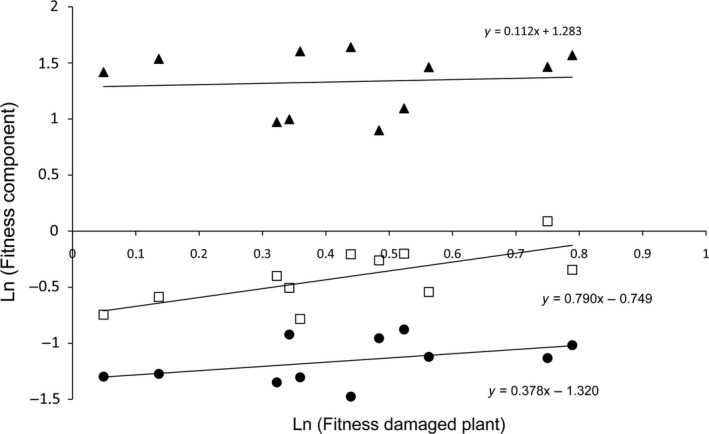
In a key‐factor analysis plant fitness (log dry weight of the damaged plants at the end of the experiment) is regressed on its five different components (Equation 6). The three components with the strongest relation with fitness (highest slope) are shown: triangles = RGR control in the second period (0.112), open squares = regrowth measure REG2 (0.790), circles fraction biomass in roots (0.378). The slopes for initial weight (−0.0971) and RGR in the first period (−0.1831) are not shown. The five slopes (Equation 6) add up to one, so that each slope shows its relative effect on variation in fitness

Using the data from Table 1 (and *W*_0_ = 0.0014 for the Dutch and *W*_0_ = 0.0015 for the US plants), fitness depends most strongly on 4REG2 (β = 0.79, *SE* = .13, *p* = .02) and the natural log of fraction biomass in roots (β = 0.38, *SE* = .26, *p* = .18) and less on the natural log of weight at the start begin (β = −0.10, *SE* = .04, *p* = .05) and on 8RGR of the control in the first period (β = −0.18. *SE* = .30, *p* = .56) or 4RGR of the control in the second period (β = 0.11, *SE* = .41, *p* = .79) period (Figure 4). We used linear regression (function lm) in the statistics program R, which generates standard errors and significance values. The procedure illustrates that we can distinguish be effects of growth, regrowth (REG2) and fraction biomass in roots on final dry weight (fitness).

## 8 Discussion

### 8.1 Time lag period

The plant will not regrow immediately but with some time lag. In this paper, growth was calculated over a period of 4 weeks, starting immediately after defoliation. We could also have waited with growth measurements until the first new leaf unfolded. We did not record this lag time, which could well be different between genotypes. Differences in growth rate of the damaged plants λ_d_ could therefore be due to a different time lag or to a difference in RGR once plants start growing again. It is recommended to keep these two parameters apart.

The commonly used tolerance measure TOL1 was higher for the Dutch than for the US populations, with the highest significance in Table 1. But this reflects two aspects of tolerance. Dutch plants showed lower shoot/root ratios. This was expected considering the long history of herbivory by the specialist *T. jacobaea* in the Dutch populations and its absence in the US populations. The regrowth ability (REG2) was also higher in the Meijendel population. Thus, both adaptations were in the same direction. Note, however, that this need not be true for REG2. If biomass loss occurs through mowing, fast regrowers almost certainly have a competitive edge. However, if the loss occurs through specialist herbivory and the same herbivore is still around 3–4 weeks after the first herbivory, the same plant could be eaten twice. In that case, there could be selection to regrow slower, to avoid the herbivore. Slow regrowth as a survival strategy is shown by the clonal species *Solidago missouriensis*. After massive herbivory different clones took between 1 and 10 years to reappear, recolonizing their habitat within a single season (Morrow & Olfelt, 2003). In this species, a long time lag for recovery is apparently a successful strategy to avoid the herbivore. REG2 and shoot–root ratio could change in different directions, and then, it makes even more sense to keep them separate in the analysis. Different changes in shoot–root ratio and REG2 will not become clear if only TOL1 is considered.

### 8.2 Costs of tolerance

As noted by Strauss and Agrawal (1999), an intuitive way to test for cost of tolerance is plot dry mass of undamaged plants *W*_c,2_ (on *x*) against fitness of damaged plants *W*_d,2_ (see also Hochwender et al., 2000). For the *J. vulgaris* data in Table 1, this gives no significant negative (*r* = −.12, *p* = .72) relationship. It seems reasonable that plants that make no investment in tolerance will suffer the greatest reduction in their growth rate (REG2 smallest). Plants with high RGR in the control are the ones that invest least in regrowth. One would then expect a negative correlation between RGR_c,1_ (or RGR_c,2_) and REG2. Neither the correlation between RGR_c,1_ and REG2 (*r* = −.39, *p* = .22) nor that between RGR_c,2_ and REG2 (*r* = −.17, *p* = .61) was significant for the *Jacobaea* data. Root storage draws resources away from primary production, and one expects this to be costly. This can be tested by plotting the fraction dry weight in the roots (1 − *h*) against the RGR of control plants. A negative relation existed in the *J. vulgaris* data between 1 − *h* and RGR_c,2_ (*r* = −.55, *p* = .07), but this correlation was almost zero for 1 − *h* and RGR_c,1_ (*r* = .05, *p* = .87). It has been emphasized (Strauss & Agrawal, 1999) that fitness variation among families and genotype may exist and makes detection of costs problematic. The existence of trade‐offs between growth, defense, and tolerance is the basis for our expectations about the performance of native and exotic plants in the defoliation experiment. Therefore, these trade‐offs and the mechanisms underlying tolerance require further detailed study.

## Conflict of Interest

None declared.

## Author Contributions

Both authors discussed these ideas at length. TdJ wrote the manuscript. TL collected the data on *Jacobaea vulgaris*.
